# Parallel roles of neuroinflammation in feline and human epilepsies

**DOI:** 10.1016/j.tvjl.2022.105912

**Published:** 2022-12

**Authors:** Sophie Binks, Simon Lamquet, Abbe H. Crawford, Alfred Meurs, Sarosh R. Irani, Akos Pakozdy

**Affiliations:** a Oxford Autoimmune Neurology Group, Nuffield Department of Clinical Neurosciences, University of Oxford, OX3 9DU, UK; b Department of Neurology, John Radcliffe Hospital, Oxford University Hospitals Foundation Trust, Oxford OX3 9DU, UK; c Department of Neurology, Ghent University Hospital, Ghent, Belgium; d Clinical Science and Services, The Royal Veterinary College, Hertfordshire AL9 7TA, UK; e University Clinic for Small Animals, University of Veterinary Medicine Vienna, Austria

**Keywords:** Autoimmune, Cats, Contactin-associated protein 2 (CASPR2), Encephalitis, Leucine-rich glioma-inactivated 1 (LGI1)

## Abstract

Autoimmune encephalitis refers to a group of disorders characterised by a non-infectious encephalitis, often with prominent seizures and surface neuronal autoantibodies. AE is an important cause of new-onset refractory status epilepticus in humans and is frequently responsive to immunotherapies including corticosteroids, plasma exchange, intravenous immunoglobulin G and rituximab. Recent research suggests that parallel autoantibodies can be detected in non-human mammalian species. The best documented example is leucine-rich glioma-inactivated 1 (LGI1)-antibodies in domestic cats with limbic encephalitis (LE). In this review, we discuss the role of neuroinflammation and autoantibodies in human and feline epilepsy and LE.

## Highlights

• Surface neuronal antibodies are a cause of limbic encephalitis (LE) in humans.
• Of these, leucine-rich glioma-inactivated 1 (LGI1) antibodies are known in feline LE.
• Affected cats present acutely with cluster focal seizures with orofacial involvement.
• Immunotherapy is effective in human cases and merits further evaluation in cats.
• A collaborative approach could yield translational benefit across the species barrier.

## Introduction

More than 50 million people worldwide have epilepsy, and the aetiology of many cases remains unknown (Husari and Dubey, 2019). Comparable veterinary data are difficult to obtain, but in the UK it was calculated 0.16 % of cats receiving veterinary care had recurrent seizures (O’Neill et al., 2020). Recently, there has been renewed attention to the role of neuroinflammation and autoimmunity in ictogenesis and epileptogenesis in animal models and human patients (Vezzani et al., 2016). In a bidirectional relationship, seizures and status epilepticus can cause neuroinflammation, and neuroinflammation and autoimmunity might promote epileptogenesis (Vezzani et al., 2016, Tan et al., 2021).

Autoimmune encephalitis (AE) refers to a group of disorders characterised by a non-infectious encephalitis, often with prominent seizures and neuronal autoantibodies (Graus et al., 2016). AE is an important cause of new-onset refractory status epilepticus (NORSE) in humans (∼37 % with surface neuronal and/or paraneoplastic antibodies in one cohort; Gaspard et al., 2015). Importantly, AE associated with surface neuronal antibodies is usually responsive to immunotherapies including corticosteroids, plasma exchange (PLEX), intravenous immunoglobulin G (IVIG; Thompson et al., 2018) and rituximab (Nosadini et al., 2021, Thaler et al., 2021, Uy et al., 2021).

It is now emerging that surface neuronal antibodies can be detected in non-human mammalian species. The best documented example is leucine-rich glioma-inactivated 1 (LGI1)-antibodies in domestic cats with limbic encephalitis (LE; Pakozdy et al., 2013). Another example is of N-methyl D-aspartate receptor encephalitis (NMDAR-Ab-E) in a polar bear from Berlin Zoo (Prüss et al., 2015). These encephalitides with cross-species applicability, and presentations with antibodies to contactin-associated protein 2 (CASPR2), a close biological partner of LGI1, will be at the core of our review.

## Human epilepsy: autoantibodies and beyond

Immune epilepsy is an under-recognised condition and its true incidence remains unclear. Epileptic disorders for which an autoimmune mechanism is postulated include NORSE (new onset refractory status epilepticus), FIRES (febrile infection-related epilepsy syndrome), DESC (devastating epileptic encephalopathy in school-aged children) and Rasmussen encephalitis. The specific cause often remains unknown (Nabbout et al., 2011, Gaspard et al., 2018).

Seizures and status epilepticus are a feature of AE (Graus et al., 2016). It remains under debate whether patients with AE can be characterised as having an ongoing tendency to seizures (i.e. epilepsy), or experience acute symptomatic seizures as part of their early disease (De Bruijn et al., 2019, Ilyas-Feldmann et al., 2021, Smith et al., 2021). However, 10–20 % of patients with new-onset epilepsy of unknown aetiology have detectable autoantibodies, suggesting that at least some may have an autoimmune aetiology (Dubey et al., 2017, McGinty et al., 2021).

In the last decades, an increasing number of cell surface (neuronal or synaptic) autoantibodies associated with AE have been identified (Ramanathan et al., 2019, Uy et al., 2021). By comparison, disorders associated with antibodies against intracellular antigens (formerly referred to as onconeural autoantibodies) have a stronger cancer association and are less immunotherapy-responsive (Lancaster and Dalmau, 2012, Binks et al., 2021b). Intracellular antigens are not accessible *in vivo* for antibody binding, and antibodies directed against them are generally considered as biomarkers and not directly pathogenic (Bien et al., 2012, Tan et al., 2021). Whereas, antibodies targeting cell-surface/synaptic antigens can bind to their target with pathogenic mechanisms including receptor blockade, cross-linking and internalisation of receptors (Table 1, Table 2, which summarise key neuronal autoantibodies associated with seizures).

**Table 1:** Clinical, demographic and oncological associations for autoantibodies targeting extracellular cell-surface/synaptic antigens with known association with seizures.

Target	Median age (range)	Sex ratio M/F	Clinical syndrome/features	Pathophysiological mechanism	Tumour association % (cancer type)	Data in veterinary patients
AMPAR (Lai et al., 2009, Laurido-Soto et al., 2019)	∼53 years (14–92 years)	1:2	LE, seizures, memory loss	Disruption of synaptic location and reduction of receptor numbers	40–60 % (SCLC, adenocarcinoma of breast, thymoma)	–
CASPR2 (Poliak et al., 2003, Poliak et al., 1999, Van Sonderen et al., 2016b, Gadoth et al., 2017)	∼66 years (25–82 years)	9:1	LE, Morvan syndrome, cerebellar ataxia, peripheral nerve hyperexcitability and neuromyotonia	Interfere with the clustering of VGKC at juxtaparanodes of myelinated axons	< 20 % (mostly thymoma, melanoma uncommon)	No epilepsy or control cats screen-positive (Pakozdy et al., 2013)
DCC (Ohkawa et al., 2013, Torres-Vega et al., 2017)	∼54 years (41–74 years) MG patients	6:1 (MG patients)	LE and neuromyotonia, (+ LGI1 or CASPR2 antibodies); MG	Inhibits interaction with Netrin-1	Thymoma (10/12 patients)	Described in a 7 year old pet cat with fatal LE (Hasegawa et al., 2019)
DPPX (Boronat et al., 2013, Tobin et al., 2014)	∼53 years (13–75 years)	1.5:1	Multifocal encephalitis with myoclonus, tremors and hyperekplexia, diarrhoea	DPPX is an auxiliary protein of Kv4.2 VGKCs whose function is disrupted	< 20 % (lymphoma)	–
GABA-A receptor (Petit-Pedrol et al., 2014, Spatola et al., 2017)	∼40 years (2 months-88 years)	1:1	Encephalitis, seizures, frequent status epilepticus, psychosis	Reduction of receptor numbers	< 20 % (thymoma)	Identified in a Cavelier King Charles dog with encephalitis (Huenerfauth et al., 2022)
GABA-B receptor (Lancaster et al., 2010, Nibber et al., 2017, Van Coevorden-Hameete et al., 2019)	∼60 years (16–77 years)	1.5:1	LE with prominent seizures	Blockade of receptor function	40–60 % (SCLC, co-existing KCTD16 seropositivity increases cancer association to 95 %)	–
GlyR (Carvajal-González et al., 2014, Crisp et al., 2019)	∼50 years (1 – 75 years)	1:1	Progressive encephalomyelitis with rigidity and myoclonus, stiff person spectrum disorder	Receptor internalisation and/or kinetic alteration	< 20 % (mostly thymoma, less frequent breast cancer, lymphoma,)	–
mGluR1 (Spatola et al., 2020)	∼55 years (43–63 years)	1.3:1	Cerebellar ataxia, seizures uncommon	Reduction of receptor numbers	< 10–20 % (lymphoma)	–
mGluR5 (Spatola et al., 2018)	∼30 years (6–75 years)	1,2:1	LE, hyperkinetic movement disorders	Reduction of total and synaptic receptors	40 – 50 % (Hodgkin’s lymphoma)	–
IgLON5 (Sabater et al., 2014, Gaig et al., 2021)	∼64 years (46–83 years)	1:1	Sleep disorder, bulbar syndrome, progressive supranuclear palsy-like syndrome. Seizures uncommon	Neurodegeneration/ tauopathy	–	–
LGI1 (Ohkawa et al., 2013, Ariño et al., 2016, Van Sonderen et al., 2016c, Binks et al., 2018b)	∼64 years (31–84 years)	2:1	LE with frequent focal seizures, characteristic FBDS, hyponatremia, neuropathic pain	Receptor internalisation, prevent docking of LGI1 to ADAM 22/23 leading to ↓ post synaptic AMPAR function	< 10 % (thymoma)	Present in 4/14 cats with LE (Pakozdy et al., 2013)
MOG (Spadaro et al., 2015, Hamid et al., 2018, Waters et al., 2020)	∼37 years (1–74 years)	1:1	Optic neuritis, transverse myelitis, ADEM, brainstem encephalitis, encephalitis	Disruption of the cytoskeletal architecture; complement activation	–	–
Neurexin 3α (Gresa-Arribas et al., 2016)	44 years (23–57 years)	1:4	Encephalitis, central hypoventilation, orofacial dyskinesias; may resemble NMDAR-Ab-E	↓ neurexin 3α and synapse creation/maturation	–	–
NMDAR (Dalmau et al., 2008, Hughes et al., 2010, Mikasova et al., 2012, Titulaer et al., 2013, Al-Diwani et al., 2019)	21 years (2 months – 85 years)	1:4	Encephalitis, psychosis, amnesia, behavioral alterations, seizures, movement disorder, autonomic dysfunction	Cross-linking and internalization of receptors; net decrease and altered distribution of NMDARs	20 – 40 % (ovarian teratoma, tumour is rare in male and females <12 years)	Diagnosed in a polar bear (Prüss et al., 2015); postulated in three dogs with meningoencephalitis of unknown origin (Stafford et al., 2019); serological but not clinical presence in six mammalian species (cats, dogs, mice, rats, baboons and rhesus monkeys;Pan et al., 2019)

ADAM22/23, disintegrin and metalloproteinase domain‐containing protein 22/23; AMPAR α‐amino‐3–hydroxy‐5–methyl‐4–isoxazolepropionic acid receptor; CASPR2, contactin-associated protein 2; DCC, deleted in colorectal carcinoma; DPPX, dipeptidyl-peptidase–like protein-6; F, female; FBDS, faciobrachial dystonic seizures; GABA-A/GABA-B, gamma-amino butyric acid A or B; GlyR, glycine receptor; IgLON5, immunoglobulin-like cell adhesion molecule 5; KCTD16, potassium channel tetramerization domain containing 16; LE, limbic encephalitis; LGI1, leucine-rich glioma inactivated 1; mGluR1 5, Metabotropic glutamate receptor 1/ 5; M, male; MG, myasthenia gravis; NMDAR, N-methyl D-aspartate receptor; SCLC, small-cell lung cancer; VGKC, voltage-gated potassium channel-complex; ↓, reduced.

**Table 2:** Clinical, demographic and oncological associations for selected antibodies targeting intracellular antigens with known association with seizures.^a^.

Target	Median age (range)	Sex ratio M/F	Clinical syndrome/features	Tumour association % (cancer type)	Data in veterinary patients
Amphiphysin (De Camilli et al., 1993, Pittock et al., 2005)	∼64 years (46–80)	1:1.5	LE, stiff-person syndrome, myelopathy, polyradiculoneuropathy	> 80 % (breast cancer, SCLC)	–
ANNA1 or anti-Hu (Graus et al., 2001)	∼63 years (28–82)	3:1	LE, cerebellar degeneration, neuropathy, autonomic dysfunction, myelopathy	> 80 % (SCLC, children - neuroblastoma)	–
ANNA2 or anti-Ri (Pittock et al., 2003, Simard et al., 2020)	∼66 years (47–87)	1:3	Cerebellar degeneration, brainstem encephalitis, opsoclonus-myoclonus	> 80 % (breast cancer, gynaecologic, SCLC)	–
CRMP5 or CV2 (Yu et al., 2001, Dubey et al., 2018b)	∼69 years (44–88)	1:1	Encephalomyelitis, cerebellar degeneration, chorea, peripheral neuropathy	> 80 % (SCLC, thymoma)	–
GAD65 (Saiz et al., 2008)	∼56 years (14–77)	1:3	LE, focal-onset seizures, stiff-person spectrum disorder	< 10 %	Antibodies in a ‘stiff dog’ and ‘stiff horse’ syndrome (Cantatore et al., 2022; Pancotto and Rossmeisl, 2017, Purcell et al., 2012)
GFAP (Fang et al., 2016, Flanagan et al., 2017, Shan et al., 2018)	∼ 44 years (8–103)	1:1	Meningoencephalomyelitis, abnormal vision, autonomic dysfunction, movement disorders; often in association with other entities e.g. NMDAR-antibodies	+ /- 30 % (ovarian teratoma)	Antibodies in pug dogs with NME (Greer et al., 2010, Pedersen et al., 2011)
KLHL11 (Mandel-Brehm et al., 2019; Dubey et al., 2020b)	41 years (27–68)	1:0	Rhombencephalitis (brainstem and/or cerebellar involvement), LE, hearing loss/ tinnitus	> 80 % (testicular germ cell tumours, primarily seminoma)	–
Ma1/Ma2 or anti-Ta (Dalmau et al., 2004, Ortega Suero et al., 2018)	60 years (18–81)	2:1	LE, diencephalic encephalitis, rhombencephalitis	> 80 % (testicular germ cell tumors, non SCLC especially + Ma1)	–
PCA1 or anti-Yo (Peterson et al., 1992))	∼61 years (26–85)	Rarely in men	Cerebellar ataxia; peripheral neuropathy, myelopathy, encephalitis all uncommon	> 80 % (breast, ovarian or fallopian tube)	–

ANNA1/ 2, anti-neuronal nuclear antibody 1 or 2; CRMP5, collapsin response mediator protein 5; F, female; GAD65;, glutamic acid decarboxylase 65; GFAP, glial fibrillary acidic protein; KLHL11, Kelch-like protein 11; LE, limbic encephalitis; M, male; MOG, myelin oligodendrocyte glycoprotein; NMDAR, N-methyl D-aspartate receptor; NME, necrotizing meningoencephalitis; PCA, Purkinje cell antibody; SCLC, small-cell lung cancer.

a These antibodies are held to be biomarkers of a predominantly T-cell-mediated cytotoxic process and separate details on pathogenic action are not provided.

## Clinical presentation

The classic presentation of human autoimmune seizures or AE is of new ASM (anti-seizure medication) -refractory seizures, frequently of temporal lobe origin, with one or more additional components of AE (Dubey et al., 2017, Husari and Dubey, 2019). The clinical course of AE has an acute to subacute progression. If untreated, it may progress to refractory status epilepticus (Graus et al., 2016). Additional manifestations of AE vary but can include acute behavioral alterations, amnesia, movement disorder, psychosis, affective disorder, sleep disturbances, and a viral prodrome (preceding symptoms similar to or suggestive of a viral infection; Uy et al., 2021). Based on specific clinical characteristics, for example the presence of faciobrachial dystonic seizures (FBDS) - a seizure type pathognomic of LGI1-autoantibodies, comprising brief unilateral contractions of the face and/or arm (and/or leg), lasting a few seconds and which can occur hundreds of times per day (Irani et al., 2011) - a certain subtype of AE can be suspected.

## NMDAR-Ab-E

NMDAR-Ab-E is one of the most common types of AE in humans (Dubey et al., 2018a) and predominantly affects children and young women. In some patients, a trigger can be identified; the presence of ovarian teratoma in ∼30 % (Dalmau et al., 2008, Titulaer et al., 2013, Al-Diwani et al., 2019) and a history of herpes simplex virus encephalitis in ∼5 % (Armangue et al., 2014, Hacohen et al., 2014).

Features indicative of a viral infection in ∼30 % may precede the onset of psychiatric symptoms which are present from an early stage and often the first manifestation (Irani et al., 2010b, Al-Diwani et al., 2019). The psychiatric presentation is characterised by spanning multiple domains including behavioural change, psychotic features (delusions, hallucinations and paranoia) and mood disturbances (Al-Diwani et al., 2019). The natural history in an individual patient is for progression to a number of other features including seizures (especially focal aware and focal to bilateral tonic-clonic seizures (Yao et al., 2019)), encephalopathy, a prominent and complex movement disorder (frequently ‘waxy’ catatonia, oral dyskinesias and choreoathetosis) and autonomic dysfunction (Irani et al., 2010b, Dalmau et al., 2011, Varley et al., 2019).

## Antibodies associated with the voltage-gated potassium channel complex (VGKC)

**Fig. 1:**
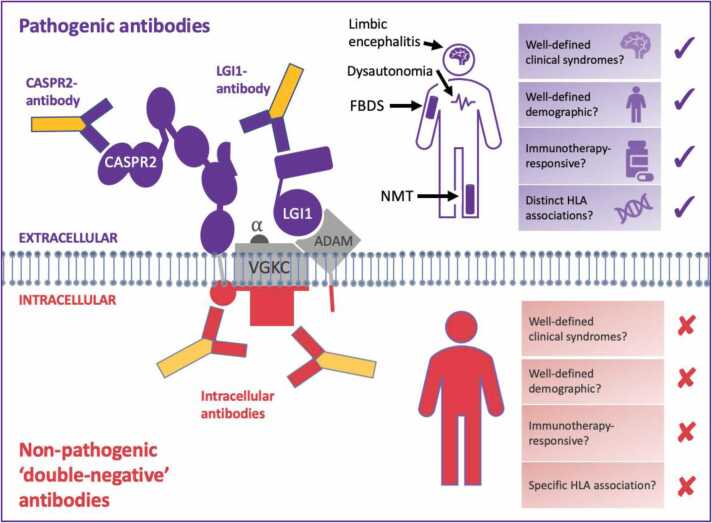
Pathogenic antibodies can target cell-surface proteins. Autoantibodies to LGI1 and CASPR2 (purple) target surface proteins and are associated with distinct clinical phenotypes. By contrast, ‘double negative’ (red) antibodies to the VGKC have intracellular targets, are unlikely to exert a biological effect in vivo and are not associated with a clear clinical syndrome. This is an open access article distributed under the terms of the Creative Commons CC BY 4.0 license, which permits unrestricted use, distribution, and reproduction in any medium, provided the original work is properly cited. CASPR2, contactin-associated protein 2; FBDS, faciobrachial dystonic seizures; HLA, human leukocyte antigen; LGI1, leucine-rich glioma-inactivated 1; NMT, neuromyotonia; VGKC, voltage-gated potassium channel.

Antibodies to the VGKC were initially described in patients with neuromyotonia, Morvan syndrome and LE (Sinha et al., 1991, Shillito et al., 1995, Buckley et al., 2001), as well as in seizuring cats (Pakozdy et al., 2013). Subsequently, it became clear that the true pathogenic mediators target the extracellular domains of VGKC-associated proteins LGI1 and CASPR2 (Irani et al., 2010a, Michael et al., 2020; Fig. 1). The remaining autoantibodies are, in fact, directed against intracellular components of the VGKC and other associated proteins, and are very likely non-pathogenic since they are not able to access their epitopes *in vivo* (Lang et al., 2017). Overall, patients with LGI1- or CASPR2-antibodies tend to be male with disease onset in late-middle age and, in both groups, additional specific features can be recognised (Binks et al., 2018a, Van Sonderen et al., 2016a, Van Sonderen et al., 2016b, Van Sonderen et al., 2016c).

Figure reproduced from Michael et al. (2020).

### LGI1-antibody encephalitis

The principal clinical features in LGI1-antibody encephalitis are seizures, cognitive impairment, and personality and behavioral abnormalities. Hyponatraemia is a frequent biochemical finding (Irani et al., 2010a, Irani et al., 2011). Subtle focal seizures and FBDS mostly occur in the acute stage and before onset of memory disturbance, and their rapid and effective treatment with immunotherapy likely abrogates the onset of cognitive decline (Thompson et al., 2018). FBDS are specific for LGI1-antibody encephalitis and depending on cohort affect up to ∼60 % of patients (Navarro et al., 2016; van Sonderen et al., 2016a, 2016b, 2016c; Gadoth et al., 2017). Paroxysmal dizzy spells – stereotyped episodes, sometimes described as intense dizziness not clearly vestibular in nature and considered a likely epileptogenic phenomenon - and autonomic (pilomotor) seizures, as well as myriad other focal seizure semiologies, are also well-recognised in LGI1-antibody encephalitis (Aurangzeb et al., 2017, Gadoth et al., 2017).

### CASPR2-antibody encephalitis

The most frequent central nervous system (CNS) syndromes associated with CASPR2-antibodies include LE (∼40 %) and Morvan syndrome (∼30 %), a disorder affecting both the peripheral and central nervous system and characterised by neuromyotonia, neuropsychiatric symptoms (especially insomnia), dysautonomia and neuropathic pain. Patients with Morvan syndrome and CASPR2-antibodies frequently have an underlying thymoma. Cerebellar ataxia is a feature in ∼35 % (Irani et al., 2010a, Irani et al., 2012; Van Sonderen et al., 2016a, Van Sonderen et al., 2016b, Van Sonderen et al., 2016c). A characteristic and intractable neuropathic pain syndrome was recently described, occurring more frequently in CASPR2- than LGI1-antibody patients (Ramanathan et al., 2021).

## Diagnostic approach and ancillary studies in autoimmune encephalitis

A high index of suspicion is required. A detailed history and physical examination are important first steps and an extensive workup then should be performed to confirm the diagnosis, but also to exclude non-inflammatory etiologies (e.g. metabolic, infectious, neoplastic; Graus et al., 2016). The 2016 consensus criteria provide a structured framework for the diagnosis of AE in humans, outlining clinical and paraclinical features required for ‘possible’ and ‘definite’ AE (Table 3, reproduced with permission), as well as consideration of seronegative cases, in which there is no identifiable autoantibody.

**Table 3:** Diagnostic criteria for ‘possible’ and ‘definite’ autoimmune encephalitis (AE). Reprinted from Graus et al. (2016), with permission from Elsevier.

Possible autoimmune encephalitis	Definite autoimmune encephalitis
Diagnosis can be made when all three of the following criteria have been met: 1. Subacute onset (rapid progression of less than 3 months) of working memory deficits (short-term memory loss), altered mental status^a^, or psychiatric symptoms 2. At least one of the following: • New focal CNS findings • Seizures not explained by a previously known seizure disorder • CSF pleocytosis (white blood cell count of more than five cells per mm³) • MRI features suggestive of encephalitis^b^ 3. Reasonable exclusion of alternative causes	Diagnosis can be made when all four^c^ of the following criteria have been met: 1. Subacute onset (rapid progression of less than 3 months) of working memory deficits, seizures, or psychiatric symptoms suggesting involvement of the limbic system 2. Bilateral brain abnormalities on T2-weighted fluid-attenuated inversion recovery MRI highly restricted to the medial temporal lobes^d^ 3. At least one of the following: • CSF pleocytosis (white blood cell count of more than five cells per mm^3^) • EEG with epileptic or slow-wave activity involving the temporal lobes 4. Reasonable exclusion of alternative causes

CNS, central nervous system; CSF, cerebrospinal fluid; EEG, electroencephalogram; MRI, magnetic resonance imaging.

a Altered mental status defined as decreased or altered level of consciousness, lethargy, or personality change.

b Brain MRI hyperintense signal on T2-weighted fluid-attenuated inversion recovery sequences highly restricted to one or both medial temporal lobes (limbic encephalitis), or in multifocal areas involving grey matter, white matter, or both compatible with demyelination or inflammation.

c If one of the first three criteria is not met, a diagnosis of definite limbic encephalitis can be made only with the detection of antibodies against cell-surface, synaptic, or onconeural proteins.

d ¹⁸Fluorodeoxyglucose (¹⁸F-FDG) PET can be used to fulfil this criterion. Results from studies from the past 5 years suggest that ¹⁸F-FDG-PET imaging might be more sensitive than MRI to show an increase in FDG uptake in normal-appearing medial temporal lobes

**Fig. 2:**
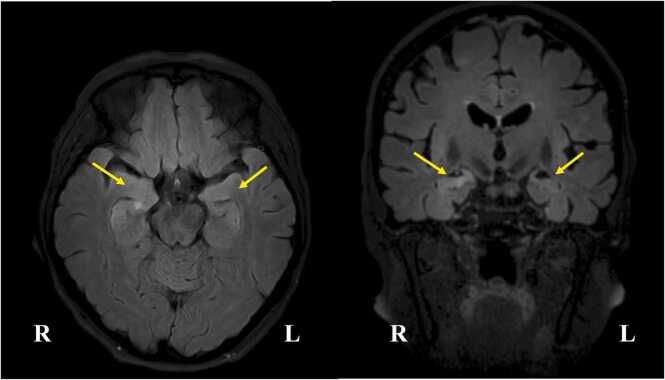
Example of a brain MRI in a human patient with LGI1-antibody encephalitis. Brain MRI in a human patient with LGI1-antibody encephalitis, showing bilateral FLAIR hyperintensity in the medial temporal lobes, most prominently on the right side (yellow arrows). Image kindly provided by Drs Simon Lamquet and Alfred Meurs. The Ethical Committee of UZ Gent has declared that there are no ethical objections to the use of anonymized MRI images. Informed consent was given by the patient. FLAIR, fluid-attenuated inversion recovery; L, left; MRI, magnetic resonance imaging, R, right.

Brain magnetic resonance imaging (MRI) is usually included in the diagnostic workup for new-onset epilepsy or encephalitis. The classical radiological features of LE are medial temporal high signal on fluid-attenuated inversion recovery (FLAIR) or T2 sequences in the acute phase (Fig. 2). Contrast enhancement has been reported in a minority of LGI1/CASPR2 patients (Kelley et al., 2017). MRI may also be normal, especially in the early stage of the disease and particularly in NMDAR-Ab-E (Irani et al., 2010b, Titulaer et al., 2013). After LGI1- or CASPR2-antibody AE, mesial temporal atrophy and (less frequently) sclerosis are typical MRI sequelae (Kotsenas et al., 2014).

CSF analysis is essential, given that inflammatory cerebrospinal fluid (CSF) may be the only abnormality found upon testing, and to exclude infection. CSF findings in AE vary and include mild to moderate lymphocytic pleocytosis, increased protein, elevated IgG index and positive (unmatched in the serum) intrathecal oligoclonal bands (OCB). CSF may also be normal and a normal CSF does not rule out a diagnosis of AE (Graus et al., 2016) and Table 3. LGI1- and CASPR2-antibody encephalitis are associated with a low frequency of pleocytosis and OCB (Blinder and Lewerenz, 2019). Specific antibody testing in humans and other mammals is discussed in detail below. Despite the ongoing discovery of novel antibodies and new techniques for detection, there is a significant group of AE patients that remains antibody-negative: which can be up to 30 % of LE in some cohorts (Graus et al., 2008). These patients may have a T-cell mediated process or harbour an as-yet unidentified antibody (Abboud et al., 2021).

Electroencephalography (EEG) is an important diagnostic tool in human AE. It may show nonspecific diffuse or focal slowing or epileptiform activity. In NMDAR-Ab-E, extreme delta brush (rhythmic delta activity at 1–3 Hz with superimposed beta bursts at 20–30 Hz) is highly characteristic, but is a late feature usually occurring after diagnosis (Irani et al., 2010b, Schmitt et al., 2012).

## Screening for an associated neoplasm

Cancer screening is recommended in patients with AE (Graus et al., 2016). The most common neoplasms associated with AE (more common with ‘onconeural’ than surface neuronal antibodies) are small cell lung carcinoma, thymoma, breast cancer, seminoma, neuroblastoma and lymphoma (Table 1, Table 2; Sechi and Flanagan, 2021). In general, a computed tomography (CT) chest, abdomen and pelvis with contrast are used as initial screening. Additional scrotal ultrasound in men and mammogram (and transvaginal sonography or pelvic MRI to search for ovarian teratoma) in women are recommended, especially when neoplasia is strongly suspected (Binks et al., 2021b). Whole body FDG-PET(fluorodeoxyglucose-positron emission tomography) can be helpful in identifying occult cancers before they clinically manifest or when initial screening with CT is negative or inconclusive (Abboud et al., 2021).

## Treatment and outcome in humans

Treatment of AE and the associated epilepsy consists of immunotherapy and symptomatic therapy, which includes appropriate anti-seizure medication. Seizures in AE are often resistant to anti-seizure medication alone. Early initiation of immunotherapy is associated with better seizure control and cognitive recovery (Thompson et al., 2018, Abboud et al., 2021). In LGI1-antibody encephalitis, ASMs alone stopped FBDS in only 10 % of patients, while addition of immunotherapy led to cessation of FBDS at one month in 51 %. Moreover, early cessation of FBDS prevented the development of cognitive impairment (Thompson et al., 2018). In humans, an elevated rate of drug-associated rash in LGI1-antibody patients, including with common antibiotics and ASMs such as carbamazepine, phenytoin and levetiracetam, is well evidenced and occurs in ∼35 % (Binks et al., 2018b).

Preferred first-line immunotherapy includes high-dose corticosteroid therapy (e.g. methylprednisolone, 1 g/day for 3–5 days), PLEX (up to 5 exchanges) and/or IVIG (total dose of 2 g/kg bodyweight given over 2–5 days; Husari and Dubey, 2019; Abboud et al., 2021). Only IVIG has randomised controlled trial (RCT) evidence (Dubey et al., 2020a), but some centres, including at Oxford, prefer PLEX, finding it more effective in clinical practice (Uy et al., 2021). When AE is associated with an underlying neoplasm, tumour therapy is crucial and associated with improved outcomes (e.g. teratoma removal in NMDAR-Ab-E; Binks et al., 2021b; Sechi and Flanagan, 2021).

Second-line immunotherapies include rituximab, a chimeric (mouse-human) monoclonal antibody to CD20, which acts via B-cell depletion. This is now proven to be a key treatment to facilitate good long-term outcomes in NDMAR-Ab-E, including relapse prevention, and is entering into more routine practice in patients with LGI1- and CASPR2-antibodies as well (Nosadini et al., 2021, Thaler et al., 2021, Uy et al., 2021). Relapse may affect ∼10 % of NMDAR-Ab-E (Nosadini et al., 2021) and ∼30 % of LGI1-antibody patients (Van Sonderen et al., 2016a, Van Sonderen et al., 2016b, Van Sonderen et al., 2016c) and is a factor in considering maintenance therapy. While long-term steroids are not advocated in NMDAR-Ab-E, in Oxford, LGI1- and CASPR2-antibody steroid-tolerating patients are often prescribed a gentle oral taper (over ∼2 years), to minimise relapse risk, although the optimal duration of maintenance therapy is unknown (Uy et al., 2021).

Residual deficits can include cognitive dysfunction, behavioral alterations and epilepsy. Hence, outcomes are far from complete. Long-term outcome is generally more favourable in AE with autoantibodies directed to extra-cellular targets rather than AE with autoantibodies to intracellular antigens. This reflects irrevocable T cell mediated tissue damage and the greater oncological association of intracellular-associated AE (Binks et al., 2021b). Nevertheless, in LGI1-antibody encephalitis, despite relatively low levels (∼20 %) of long-term moderate to severe disability in most studies, ∼60 % are impaired on at least one of cognition, mood or fatigue (Finke et al., 2017, Binks et al., 2021a). The overall risk of secondary or chronic epilepsy varies with the target antigen. Patients with NMDAR-Ab-E rarely develop chronic epilepsy, but there is a higher chance of residual deficits and seizures when the AE is triggered by herpes simplex encephalitis (Armangue et al., 2018). In one study, 85 % of patients with LGI1-antibody encephalitis were seizure free two years after onset, and only 14 % were still taking anti-seizure medications (van Sonderen et al., 2016a, 2016b, 2016c) but other investigators have outlined a ∼20 % chance of epilepsy in the chronic phase of this condition (Smith et al., 2021).

## Autoantibody detection in human and veterinary patients

**Fig. 3:**
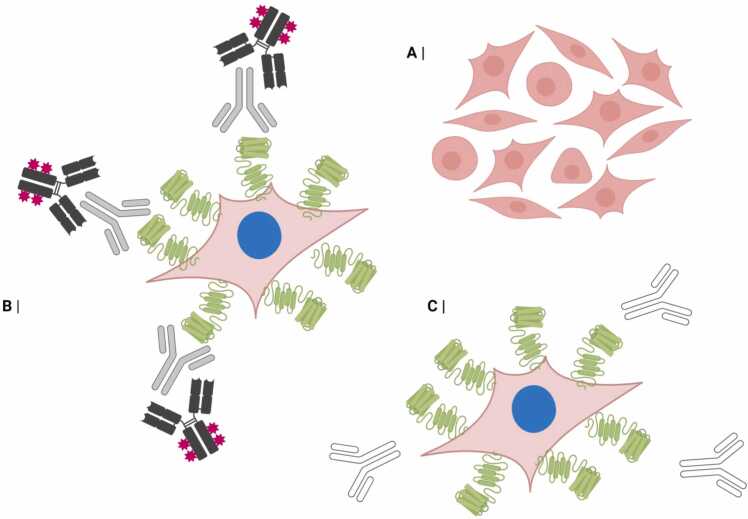
Schematic representing a live cell-based assay. Cells in culture (A) are transfected with DNA to express the antigen of interest tagged with a green EGFP protein (B and C). Pathogenic autoantibodies bind to the expressed antigen and are then labelled with a fluorophore-labelled secondary antibody (B). In (C) non-antigen specific antibodies do not bind and are washed away. Image created in Biorender. DNA, deoxyribonucleic acid; EGFP, enhanced green fluorescent protein.

**Fig. 4:**
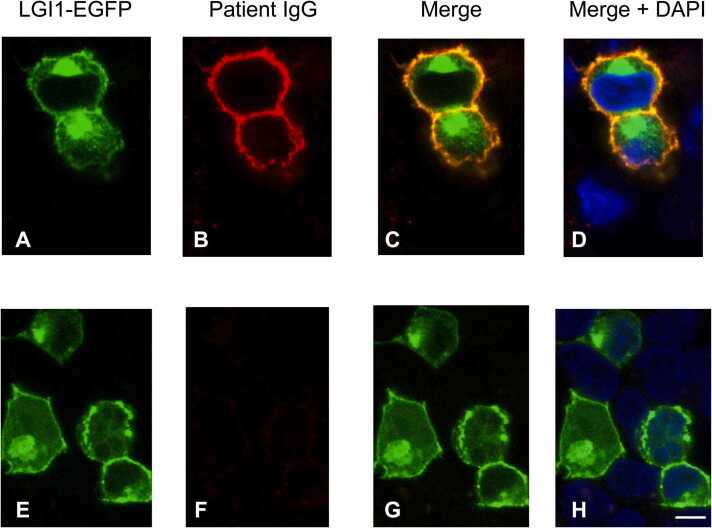
Cell-based assay of a 2-year-old male neutered Bengal cat with seizures and LGI1 antibodies. Cells expressing feline LGI1 (A) are bound by cat IgGs tagged with feline-specific red-fluorescent secondary antibody to feline IgG (B) which co-localizes under fluorescence microscopy (C). Panel D additionally depicts nuclei stained with DAPI, showing no feline serum binding to a non-transfected cell. Panels E-H show the same sequences in a control epilepsy cat demonstrating a negative result. Scale bar on panel H represents 10 µm. Ethical approval was granted by the Royal Veterinary College Clinical Research Ethical Review Board (CRERB; Approval number, URN 2020 1957–2; Approval dates, 6 April 2020 and 12 May 2021). DAPI = r 4′,6-diamidino-2-phenylindole; IgG, immunoglobulin; LGI1, leucine-rich glioma-inactivated 1.; EGFP, enhanced green fluorescent protein.

The central method of surface neuronal antibody detection in humans and other mammals is the cell-based assay (CBA). In this technique, the antigen/epitope of interest is expressed on the extracellular aspect of HEK (human embryonic kidney) 293 T cells (an immortalised cell line). Patient (human or non-human) serum or CSF is applied and incubated with the transfected cells. If the sample harbours autoantibodies to the extracellular domain of the over-expressed protein, it can be detected with a fluorophore-labelled secondary antibody. Co-localisation of the antigenic target (often EGFP (green fluorescent protein) labelled) and autoantibodies is visible under fluorescence microscopy (Irani et al., 2010a; Fig. 3, Fig. 4).

In commercial laboratories, most CBAs are performed using cells which are preservative-fixed and immobilised. This may alter the conformation of expressed antigen and/or partially permeabilise the membrane exposing intracellular proteins not ‘visible’ in a physiological context (Michael et al., 2020). International studies using blinded samples have demonstrated a reduced accuracy of fixed methods when compared to assays which maintain physiological conditions. These include the ‘live’ CBA, where the cells are maintained alive in culture prior to staining or fluorescence-activated cell sorting (FACS; Waters et al., 2012). A recent comparison of fixed kits versus research live assays concluded false negatives to be a significant issue for several antigens: LGI1-antibody samples were among the most likely to be affected, with a false negative rate of 17 % (11/63; Ruiz-García et al., 2021).

CSF screening is usually recommended in addition to serum in most cases. However, sensitivity differs between the antigenic targets; in LGI1-antibody encephalitis serum antibodies are more sensitive and CSF antibodies detected in around ∼40 % of human patients (Van Sonderen et al., 2016a, Van Sonderen et al., 2016b, Van Sonderen et al., 2016c), whereas in NMDAR-Ab-E the presence of CSF antibodies is critical and forms a part of diagnostic criteria (Graus et al., 2016). Serum and/or CSF IgG antibody titres generally have a limited predictive value for treatment response or relapse rate and the meaning of persistent seropositivity in (frequently recovered) human and feline patients is an area of active enquiry.

For veterinary patients there is an additional factor. The above assays were developed to detect antibodies in human patients and are not specialised to veterinary cohorts. The importance of species-specific testing was recently highlighted in canine glial fibrillary acidic protein (GFAP)-antibody meningoencephalitis, a well-described entity in pug dogs with a certain dog leucocyte antigen (DLA) haplotype (Greer et al., 2010, Pedersen et al., 2011). Investigators screened a number of dogs with a suggestive phenotype using CBA and tissue-based immunohistochemistry on rodent brain sections, as used in human diagnosis, and the failure to detect any positive cases was thought to be at least in part ascribable to species-specific differences in epitopes (Rozental et al., 2021). Therefore, veterinarians suspecting autoantibody-mediated LE are advised to liaise with research laboratories with an interest and experience in studying non-human cases. Our laboratory research programme is working to optimise assays and protocols for feline patients, and our observations concur with those of Rozental et al. (2021) that human assays require adaptation for the target mammalian group.

So far, LGI1-autoantibodies - but not CASPR2-autoantibodies - have been discovered in cats with LE (Pakozdy et al., 2013). The clinical phenotype, paraclinical investigations (MRI and EEG where available) and neuropathology findings of feline LGI1-autoantibody cases closely parallels that of human patients (Pakozdy et al., 2013, Pakozdy et al., 2014, Klang et al., 2014). In fact, the clinical presentation of cats with spontaneously-arising LGI1-autoantibodies recapitulates features of human disease more comprehensively than do available mouse models (Petit-Pedrol et al., 2018).

NMDAR-Ab-E is yet to be clinically reported in domestic cats. One laboratory-based study screened 71 cat sera from a small animal hospital outpatient clinic and reported up to 37 % seroprevalence of NMDAR-antibodies in older animals (aged 12–22 years). The authors found similar results in dogs, rats, mice, baboons and rhesus macaque monkeys. The assays were performed using fixed kits and CSF in these species was not tested (Pan et al., 2019). This is relevant because in humans, community prevalence studies show NMDAR-antibodies in up to ∼5 % of healthy and disease control sera (Dahm et al., 2014) and CSF antibodies are part of diagnostic criteria for NMDAR-Ab-E (Graus et al., 2016). Additionally, further clinical information on these cats would be important to assess the biological role of the detected antibodies.

In terms of symptomatic veterinary patients, the polar bear Knut is a suspected NMDAR-Ab-E case. Knut had an onset of seizures in 2011, fell into the water feature of his enclosure at Berlin zoo and drowned. Pathological examination of his brain showed features consistent with encephalitis and CSF testing was positive at high titre (> 1:1000) for NMDAR-autoantibodies. Supportive evidence was also provided by immunofluorescence staining of Knut’s CSF on rodent hippocampus and cerebellum sections, which displayed a pattern comparable to that of human NMDAR-Ab-E patients (Prüss et al., 2015).

Subsequently, CSF NMDAR-antibodies have been published in 3/32 dogs with mainly meningoencephalitis of unknown origin, using a fixed assay. It was acknowledged that the binding in these cases was less striking than that seen with human CSF (Stafford et al., 2019). Whether this relates to species-specific testing factors or aspects of the fixed kit remains to be determined. However, the principal described epitope of NMDAR-antibodies, the N368/G369 residue, is very highly conserved across multiple mammalian species, including dogs, cats and polar bears (Prüss et al., 2015, Stafford et al., 2019). This indicates its functional importance and from an evolutionary perspective is in keeping with a shared disease process.

**Fig. 5:**
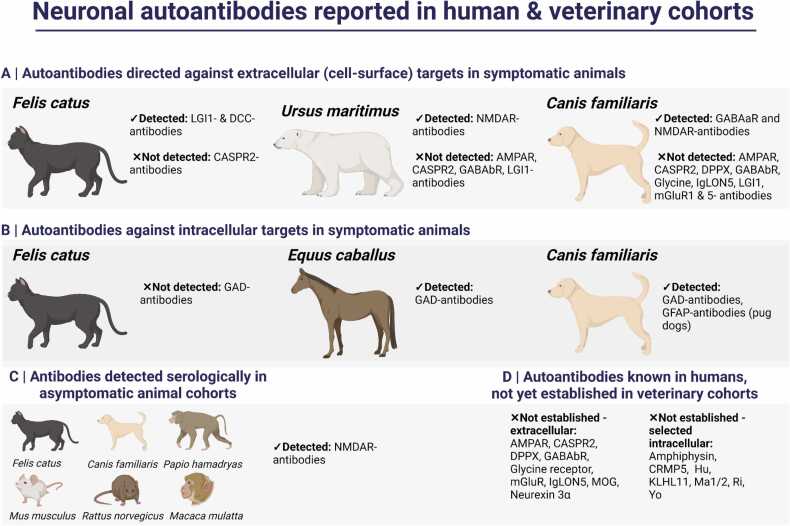
Human autoantibodies, also recognised in veterinary cohorts (A) Cell-surface antibodies detected in symptomatic domestic cats (*Felis catus*), a polar bear (*Ursus maritimus*) and domestic dogs (*Canis familiaris*). Also given are those antibodies which were screened but not yet detected in these species. (B) Intracellular antibodies detected in symptomatic horses (*Equus caballus*) and a domestic dog (GAD) with stiff horse/dog syndrome, and pug dogs with meningoencephalitis (GFAP). To date, GAD-antibodies were not found in tested domestic cats. (C) NMDAR-antibodies have been found on screening by fixed assay in asymptomatic domestic cats, dogs, baboons (*Papio hamadryas*), mice (*Mus musculus*), rats (*Rattus norvegicus*) and Rhesus macaque monkeys (*Macaca mulatta*). (D) Selected extra- and intra-cellular antibodies established in human but not veterinary patients. Created with BioRender.com. AMPAR, α‐amino‐3–hydroxy‐5–methyl‐4–isoxazolepropionic acid receptor; CASPR2, contactin-associated protein 2; CRMP5, collapsin response mediator protein 5; DCC, deleted in colorectal carcinoma; DPPX, dipeptidyl-peptidase–like protein-6; GAD, glutamic acid decarboxylase 65; GFAP, glial fibrillary acidic protein; GABAa/bR, gamma-amino butyric acid A/B receptor; GAD, LGI1, leucine-rich glioma-inactivated 1; IgLON5, immunoglobulin-like cell adhesion molecule 5; KLHL11, Kelch-like protein 11; mGluR, Metabotropic glutamate receptor; MOG, myelin oligodendrocyte glycoprotein; NMDAR, N-methyl D-aspartate receptor.

In summary, testing for neuronal surface autoantibodies in humans and other mammals is an effective diagnostic tool but must be interpreted in the setting of the type of test used, its species applicability and the clinical presentation. So far, LGI1-autoantibodies in domestic cats with LE are the best established patient group in veterinary medicine. Yet around half of suspected LE cases test negative for LGI1-autoantibodies, suggesting these cats may harbour other, as yet unidentified, specificities (Binks, Crawford, Irani, Pakozdy and collaborators, unpublished observations). For example, one cat with LE that tested negative for LGI1-antibodies but positive for antibodies to deleted in colorectal carcinoma (DCC), an axonal guidance protein, has been reported (Hasegawa et al., 2019). These antibodies were previously described in humans with LE, alongside LGI1-antibodies (Ohkawa et al., 2013). A visual summary of currently assessed autoantibodies in veterinary cohorts is provided in Fig. 5.

## The feline patient

In 2013, we described a small cohort of epileptic cats seropositive for VGKC antibodies, thus raising awareness that immune-mediated LE may exist as a naturally occurring disease in cats (Pakozdy et al., 2013). Cats with acute-onset cluster seizures (FEPSO - feline complex partial cluster seizures with orofacial involvement) were recruited prospectively into the study, and 5/14 (36 %) had elevated concentrations of VGKC-complex antibody; none harboured CASPR2-antibodies, but four also had antibodies to LGI1. This finding had important implications for veterinary medicine; detection of antibodies against CNS antigens could offer a novel means of early and accurate diagnosis of LE. Furthermore, immunosuppression could be considered as an additional treatment modality (alongside standard anti-seizure medications) to potentially optimise clinical outcomes for affected cats.

Continued investigation and characterisation of LE in cats has been hindered by the lack of a widely available serological test. Where testing of serum from selected epileptic cats has been performed, the results were available weeks or even months after the acute phase of the illness, delaying diagnosis and initiation of appropriate therapy. As continued research in human medicine revealed that seropositivity to the VGKC-associated protein LGI1 was of major pathological significance, specific testing for LGI1-autoantibodies in cats is underway to determine its translational importance.

**Fig. 6:**
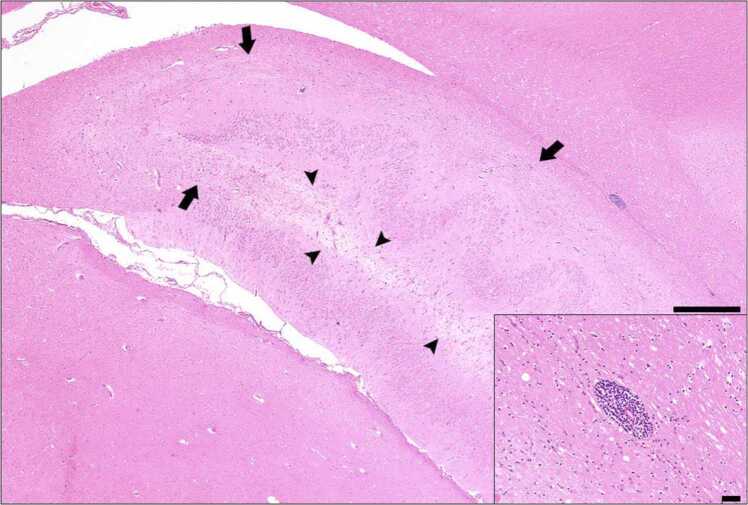
Formalin fixed paraffin embedded brain section from a 4-year-old male neutered domestic short hair cat positive for LGI1 autoantibodies. The cat was euthanized following recurrent seizures that were refractory to oral phenobarbital, levetiracetam and prednisolone. The main image (haematoxylin and eosin stain) shows hippocampus with severe neuronal loss in the CA1, CA2 and CA3 regions (arrows) and marked vacuolization, pallor and microgliosis in the hilus and CA3 regions (arrowheads), bar = 500 µm. Small image: Alveus region blood vessel showing severe vasculitis with mural and perivascular infiltration of lymphocytes and plasma cells, bar = 50 µm. Image kindly provided by Dr Alexandros Chardas, Royal Veterinary College. LGI1, leucine-rich glioma-inactivated 1.

Feline hippocampal necrosis, of which the cardinal pathological features are neuronal loss, gliosis, capillary proliferation and perivascular cuffing, sometimes with immune cell infiltrates, is described in the veterinary literature in necropsies of seizuring cats (Fatzer et al., 2000, Pakozdy et al., 2011, Wagner et al., 2014). The cause of the seizures may be infectious, toxic, metabolic, neoplastic, inflammatory or occult in some cases, and it has been debated whether the tissue damage is secondary to seizure-related excitotoxicity or a primary disease manifestation (Fatzer et al., 2000, Wagner et al., 2014). Accordingly, alongside efforts to identify autoantibodies in blood, histopathology has been performed on a small number of VGKC/LGI1-autoantibody positive cat brains. The main abnormality detected was neuronal loss in the hippocampi, usually distributed bilaterally and detected in one or more hippocampal subfields. Extrahippocampal changes in the temporal cortex, basal nuclei, subiculum and enthorrhinal cortex were additionally detected in some cats. Perivascular cuffs and/or diffuse infiltration of inflammatory cells (predominantly T cells) were present (example shown in Fig. 6) but milder than is typically seen in human counterparts. Diffuse immunoglobulin immunoreactivity in the brain parenchyma was seen in neurons, astrocytes, oligodendrocytes and microglia. Interestingly, complement deposition was detected on neuronal surfaces of seropositive cats, but not in other encephalitic cases nor healthy controls (Klang et al., 2014). Subsequent investigations have suggested that the immunoglobulin and complement deposition on neuronal surfaces is not associated with lymphocyte infiltrates (Tröscher et al., 2017), consistent with CSF samples from LGI1-antibody positive cats being typically unremarkable. Future such feline cohorts would benefit from serological testing to evaluate in more detail the relationship of antibody-status to hippocampal pathology.

The majority of LGI1-antibody positive cats present with acute-onset temporal lobe seizures. Feline temporal lobe seizures are usually characteristic in their phenotype; the main features include peri-ictal behavioural arrest, orofacial twitching, oral automatisms, mydriasis, salivation, vocalization and loss of environmental awareness. Secondary generalization is not uncommon. It is important to emphasise that while LE is a cause of temporal lobe seizures, numerous other causes have also been reported including vascular, systemic, metabolic, neoplastic and toxic aetiologies (Kitz et al., 2017). Therefore, characterization of the precise clinical phenotype, alongside appropriate diagnostic investigations are required to exclude other causes of temporal lobe seizures. In feline LE, there appears to be a tendency for cluster seizures and, less commonly, status epilepticus. However, we have not identified a specific seizure phenotype unique to LGI1-antibody positive cats. FBDS, which in humans are pathognomic of LGI1-antibody encephalitis, have not been observed in cats. And while temporal lobe seizures are a shared feature of both cats and humans with LGI1-antibodies, it is not known whether certain characteristic focal semiologies noted in humans, such as sensory and dysautonomic seizures, or paroxysmal dizzy spells, can affect feline patients. Interictal behavioural changes were reported by owners and veterinarians in a study of 17 cats with a FEPSO phenotype but antibody-status was unknown (Pakozdy et al., 2011) and further investigation is under way (Binks, Crawford, Irani, Pakozdy and collaborators).

**Fig. 7:**
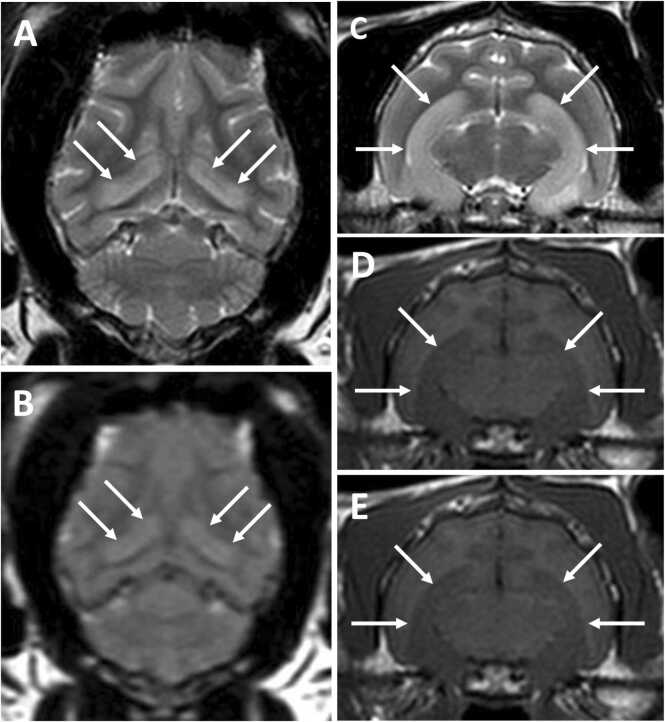
Magnetic resonance images of the head of a 5-year-old female neutered domestic short hair cat diagnosed with FEPSO and positive for LGI1-autoantibodies. (A) T2W dorsal; (B) T2W FLAIR dorsal; (C) T2W transverse; (D) T1W pre-contrast; and (E) T1W post-contrast transverse images are shown at the level of the hippocampus. White arrows highlight the diffuse T2W and FLAIR hyperintense, T1W isointense (compared to normal grey matter) signal of the hippocampus bilaterally with very mild contrast enhancement, best appreciated in the original DICOMS (E). These signal changes may represent inflammation and/or postictal oedema. Images courtesy of Dr Abbe Crawford. FEPSO, feline complex partial cluster seizures with orofacial involvement; FLAIR, fluid-attenuated inverse recovery; LGI1, leucine-rich glioma-inactivated 1; T1/2 W – T1/T2 weighted. The right side of the brain is shown on the left of the image. Slice thickness 3 mm. Dorsal sequences were obtained perpendicular to the long axis of the hippocampus (Rusbridge et al., 2015).

Typically, no relevant changes are detected on haematology, biochemistry (in contrast to the hyponatraemia recognised in humans), serological testing for feline leukaemia virus (FeLV) and feline immunodeficiency virus (FIV), thoracic radiology and abdominal ultrasonography. On brain MRI, some cats with LGI1-antibodies show hyperintensity of the hippocampi on T2W and FLAIR images, with or without associated contrast enhancement (Fig. 7) (Pakozdy et al., 2013, Pakozdy et al., 2014). The proportion of cats with abnormal brain imaging could be higher as availability and timing of scanning is likely to vary between referral centres (Binks, Crawford, Irani, Pakozdy and collaborators, unpublished observations). It should be noted that radiological assessment of the feline hippocampus remains challenging (Claßen et al., 2016) and debate ongoing as to whether signal change could represent ictal oedema or a more specific pathology (Wahle et al., 2014). However, one study suggested cats with a ‘FEPSO’ phenotype displayed more convincing radiological abnormality in limbic regions than those with other forms of epilepsy (Claßen et al., 2016). This may be consistent with a disease-specific cause, but requires clarification with antibody testing in the future. Serial MRI studies have not yet been performed to evaluate lesion development or progression.

EEG is only occasionally performed in cats. To the best of our knowledge, there is only a single published case report of EEG-confirmed epileptic activity in a cat with VGKC-antibody associated LE. The cat was clinically asleep and no motor signs were recorded during the episode, which displayed synchronous discharges. Rhythmic spike activity was detected at about 8 Hz. The spikes propagated towards the anterior leads (Pakozdy et al., 2014). Akin to humans, feline LGI1-antibody LE does not appear to be a paraneoplastic disorder. However, a single case was concurrently diagnosed with pulmonary adenoma (Klang et al., 2014).

Based on our experiences to date, it is not possible to differentiate between seropositive LE and seronegative temporal lobe epilepsy based on the above diagnostic tests, and hence in the future serological testing will be required to reach a definitive diagnosis.

Treatment of feline LE typically entails anti-seizure medications alongside supportive or intensive care. Limited data are currently available to guide clinical decision making and provide insights on expected response times and long-term prognosis. At the authors’ clinics, a combination of phenobarbital and levetiracetam is preferred, with a midazolam infusion if necessary in the acute setting. To date, there is no clear consensus or evidence on the use of prednisolone but we typically initiate prednisolone therapy (starting at 0.5–1 mg/kg/q12h for 7 – 14 days, with subsequent taper over months depending on response) if there is a poor response to anti-seizure medications An example regimen is shown in Table 4. Immunotherapy treatments routinely used in humans with LE, specifically PLEX and IVIG, have not yet been used in cats but may warrant consideration, particularly in cases showing an inadequate response to steroid treatment. In dogs with necrotizing meningoencephalitis, benefit was shown in one study by adding ciclosporin to prednisolone (Jung et al., 2007). Evaluation of ciclosporin’s efficacy in FEPSO cats, and of potential interaction with concurrent prednisolone and phenobarbital therapy, remains to be explored.

**Table 4:** Example oral steroid treatment and epilepsy medication regimen post-acute admission in cats with suspected or detected LGI1-autoantibodies.

	Prednisolone	Anti-seizure medications
1.	Prednisolone 0.5–1 mg/kg q12h for 7–14 days based on clinical response.	Phenobarbital 2–4 mg/kg q12h (add 20 mg/kg levetiracetam q8h if poor seizure control).
2.	Prednisolone 1 mg/kg q24h for 14–28 days.	Assess phenobarbital serum levels after 14–21 days of treatment. Maintain phenobarbital (and levetiracetam when used) if adequate seizure control. Increase phenobarbital dose if poor seizure control.
3.	Prednisolone 0.5 mg/kg q24h for 14–28 days.	
4.	Prednisolone 0.25 mg/kg q24h for 14–28 days.	Reassess serum phenobarbital level 14 days after dose increase. Further incremental increases in dose can be trialled if seizure control remains poor. Consider levetiracetam dose increase to 25 mg/kg q8h if ongoing poor seizure control.
5.	Prednisolone 0.25 mg/kg q48h for 28 + days. Consider stopping if seizure free, alternatively maintain low dose alternative day therapy long-term if well tolerated.	Re-assess serum phenobarbital level, haematology and biochemistry every 3–6 months. Discontinue levetiracetam after 3 months of seizure freedom.

The long-term outcome for LGI1-antibody positive cats is highly variable. In our experience, the majority of cases have responded well to treatment and have a good long-term outcome, but resistant cases also occur in which seizure activity persists despite medication. If a positive response to treatment is seen in the first month following diagnosis, the long-term outcome is typically good and seizure freedom can frequently be achieved. Studies evaluating the potential long-term consequences of LE, such as behavioural changes or cognitive deficits, have not yet been performed.

## Conclusions

Recent years have seen a rapid increase in knowledge of neuroinflammation and autoantibodies implicated in encephalitis and epilepsy in human and feline patients. LE and epilepsy associated with LGI1-autoantibodies are an evolving entity in domestic cats (Pakozdy et al., 2013), to date largely mirroring observations in humans. Further investigation will help reveal further clinical characteristics and optimal management strategies for these cats, in whom current therapeutic options are limited compared to human counterparts. This spontaneously-arising feline example of a rare disease offers the opportunity for human and veterinary medics to work together to discover mechanisms and effective treatments to the mutual benefit of both species. Similar situations may arise in other autoimmune categories and challenge the dogma of human exceptionalism and traditional approaches towards animal models, and support the ‘One Health’ ethos (Devinsky et al., 2018).

## Conflict of interest statement

SB is supported by the Wellcome Trust and has had salary support from the National Institute for Health Research (NIHR). SB holds grants from PetSavers (03.20) and 10.13039/100009449Petplan Charitable Trust (S20-924-963) and is a co-applicant on PetSavers grant MDR 12.22. SB is a co-applicant on a patent application entitled ‘Diagnostic Strategy to improve specificity of CASPR2 antibody detection’ (TBA / BB Ref. JA94536P.GBA)’. SL received no financial support for the research, authorship, and/or publication of this article. No conflicts of interest to report. AHC is a co-applicant on grants from PetSavers (03.20 and MDR 12.22) and Petplan Charitable Trust (S20-924-963). AHC is supported by the Animal Care Trust (1185 1718) at the Royal Veterinary College and Action Medical Research. AM has received speaker fees and travel grants from UCB and travel expenses from Medtronic. SRI is supported by 10.13039/100004440Wellcome Trust (Grant number 104079/Z/14/Z), a Medical Research Council Fellowship (MR/V007173/1), the BMA Research Grants- Vera Down grant (2013) and Margaret Temple (2017), 10.13039/501100000295Epilepsy Research UK (P1201), the Fulbright UK-US commission (MS-Society research award), and by the NIHR Oxford Biomedical Research Centre. The views expressed are those of the author(s) and not necessarily those of the NHS, the NIHR or the Department of Health. For the purpose of Open Access, the author has applied a CC BY public copyright licence to any Author Accepted Manuscript version arising from this submission. SRI is a co-applicant and receives royalties on a licenced patent application WO/210/046716 (U.K. patent no., PCT/GB2009/051441) entitled 'Neurological Autoimmune Disorders' and ‘Diagnostic Strategy to improve specificity of CASPR2 antibody detection. (PCT/G82019 /051257) SRI has received honoraria / research support from UCB, Immunovant, MedImmun, Roche, Cerebral therapeutics, ADC therapeutics, Brain, CSL Behring, UCB and ONO Pharma. SRI is a co-applicant on grants from PetSavers (03.20) and Petplan Charitable Trust (S20–924–963) and holds a PetSavers grant MDR 12.22. AP is a co-applicant on grants from PetSavers (03.20 and MDR 12.22) and Petplan Charitable Trust (S20–924–963). None of the authors has any other financial or personal relationships that could inappropriately influence or bias the content of the paper.
